# Case of Ascites, Cured Spontaneously

**Published:** 1819-04-01

**Authors:** E. Colville

**Affiliations:** Surgeon, Ayton, N. B.


### VIII.

*Case of Ascites, cured spontaneously.* Communicated by E. Colville, Surgeon, Ayton, N. B.

On the 22d of December, 1816, I was called to Eyemouth to visit Robert Mason, who was represented to be afflicted with strangury. On my arrival, I found him labouring under ascites, which he stated to be of two months' duration, and to have been ushered in by "a cold," caught by sitting for several hours in a damp out-house.

This person had been a seaman in His Majesty's service, and informed me, that while employed off Toulon, he received a blow which fractured his clavicle, besides considerably injuring his head. After this unlucky occurrence he never regained his wonted health and strength; nevertheless, on his return home to Eyemouth, he was able to work at his trade (that of a shoemaker) until the latter end of October; that a cold, caught in the manner above described, farther shattered his constitution, and brought on dropsy. The disease, however, was confined to the abdomen, for there was no anasarca.

At my first visit, I found his abdomen full and tense; (he said it had rapidly attained its present size) his thirst great; his urine scanty and high coloured; appetite bad, and the emaciation extreme. So far as I could judge, his liver was sound. It being evident that nothing could relieve him but the immediate operation of tapping, I performed it that same evening. His pulse, before the operation, was quick and feeble; but, upon the whole, he stood it well.

I prescribed a pill, consisting of calomel, digitalis, and pulvis ipecac. compos. which he took every day for three weeks, but without the least apparent benefit. His abdomen gradually swelled again, and rendered the operation of tapping again necessary. The medicines usually ordered under such circumstances appearing to have no effect, I had no other alternative but to have recourse to tapping as often as the tension of the abdomen rendered it necessary. Indeed, each time he himself urged the operation, saying he could bear the distension no longer. He was tapped in all eighteen times! The following is a list of the dates:— December 22d, 1816; January 2d, 18th, 31st; February 18th; March 12th, 22d; April 3d, 15th, 29th; May 13th, 29th; June 16th, 30th; July 16th, 30th; August 14th, 31st, 1817.

The water drawn off at each time was not measured; but I am sure I do not over-rate, when I say it averaged from 14 to 18 quarts.

He was occasionally affected with vomiting; and he remarked, that when this was frequent, the size of his abdomen increased more slowly. This circumstance sufficiently accounts for the difference in the length of the intervals betwixt some of the operations.

Latterly, I had not been in the habit of calling on him, (as he lives at a distance) except when the operation of tapping was to be performed; for which purpose he always sent me a message. The usual interval from the last tapping having more than elapsed, I was surprised at not hearing from him, as usual, and therefore took an early opportunity of calling on him. I was astonished to find that the ascites had disappeared, and that, though still very weak, his urine and thirst were natural. On examining him particularly, I got information, that immediately before the dropsical collection disappeared, his feet swelled, and a profuse perspiration broke out "all over him," which lasted for three days. To this circumstance, I had no hesitation in ascribing his recovery—I may truly say *unexpected* recovery—though he himself attributed it to a dose of flowers of sulphur, which he had accidentally taken; and still potently believes in the marvellous power of that drug.

He has continued free from dropsical symptoms ever since. Being only forty years old, I have no doubt he might have a perfect recovery from his present state of debility, had he but a generous diet. But he is in abject poverty, and has not the means of procuring the common comforts of life

